# Nitrogen Footprint of a Recycling System Integrated with Cropland and Livestock in the North China Plain

**DOI:** 10.3390/plants11070842

**Published:** 2022-03-22

**Authors:** Hailun Du, Jixiao Cui, Yinan Xu, Yingxing Zhao, Lin Chen, Zhejin Li, Peng Sui, Wangsheng Gao, Yuanquan Chen

**Affiliations:** 1 College of Agronomy and Biotechnology, China Agricultural University, Beijing 100193, China; helen_du@cau.edu.cn (H.D.); xuyinan@cau.edu.cn (Y.X.); yingxingzhao@alu.cau.edu.cn (Y.Z.); chenlin@cau.edu.cn (L.C.); lizhejincau@163.com (Z.L.); suipeng@cau.edu.cn (P.S.); 2 Institute of Environment and Sustainable Development in Agriculture, Chinese Academy of Agricultural Sciences, Beijing 100081, China; cuijixiao@caas.cn

**Keywords:** recycling agriculture, nitrogen footprint, waste management, crop-swine integrated system, North China Plain

## Abstract

Nitrogen-based pollution from agriculture has global environmental consequences. Excessive use of chemical nitrogen fertilizer, incorrect manure management and rural waste treatment are key contributors. Circular agriculture combining cropland and livestock is an efficient channel to reduce the use of chemical nitrogen fertilizers, promote the recycling of livestock manure, and reduce the global N surplus. The internal circulation of organic nitrogen resources in the cropland-livestock system can not only reduce the dependence on external synthetic nitrogen, but also reduce the environmental impacts of organic waste disposal. Therefore, this study tried to clarify the reactive nitrogen emissions of the crop-swine integrated system compared to the separated system from a life cycle perspective, and analyze the reasons for the differences in nitrogen footprints of the two systems. The results showed that the integrated crop production and swine production increased the grain yield by 14.38% than that of the separated system. The nitrogen footprints of crop production and swine production from the integrated system were 12.02% (per unit area) and 19.78% lower than that from the separated system, respectively. The total nitrogen footprint of the integrated system showed a reduction of 17.06%. The reduction was from simpler waste manure management and less agricultural inputs for both chemical fertilizer and raw material for forage processing. In conclusion, as a link between crop planting and pig breeding, the integrated system not only reduces the input of chemical fertilizers, but also promotes the utilization of manure, increases crop yield, and decreases environmental pollution. Integrated cropland and livestock is a promising model for agriculture green and sustainable development in China.

## 1. Introduction

Nitrogen fertilizer plays an irreplaceable role in guaranteeing food security in China. China is the country with the highest use of nitrogen fertilizer, but the utilization efficiency of chemical nitrogen fertilizers has been lower than the world average in the past 20 years [1]. The problem of nitrogen balance in the agricultural ecosystem has been widely concerned, about 200 million tons of reactive nitrogen (Nr: all species of N except N_2_) resource is lost to the environment per year as Nr and N_2_ at present [2]. A large amount of Nr enters the environment, which changes the balance state of the nitrogen cycle in the global ecosystem, and brings serious environmental problems such as surface water eutrophication, groundwater nitrate pollution, ozone layer destruction, and acid rain [3,4,5]. Measures need to be taken to transform the nitrogen cycle into a sustainable, pollution-free, and profitable model.

With the improvement of the living standards of residents, the consumption demand for livestock products is rising. The large-scale development of livestock and poultry breeding not only meets people’s increasing demand for livestock and poultry, but also produces a large number of feces, wastewater, and other wastes, which poses certain threats to soil, atmosphere, and water resources. As reported, the annual amount of livestock and poultry manure in China was 3.16 billion tons [6]. However, the comprehensive utilization rate of livestock and poultry manure in China was lower than 75% [7]. These unused agricultural wastes have caused a series of environmental pollution problems and become a thorny ecological and environmental problem in the whole world. The total amount of animal organic fertilizer (livestock and poultry manure) currently produced in China is about 14 million tons of pure nitrogen, which is equivalent to about half of the annual production of chemical nitrogen fertilizer [8]. Therefore, the resource utilization of livestock and poultry breeding waste is not only the development direction of livestock and poultry breeding waste treatment at present, but also an important way to solve livestock and poultry breeding pollution.

Therefore, an important way to solve the above problems is the cycle of planting and breeding, which not only reduces the input of chemical fertilizers, but also improves the utilization efficiency of livestock and poultry manure, and reduces the environmental pollution of livestock and poultry manure [9,10]. The development of circular agriculture with efficient recycling of resources and ecological and environmental protection as the core, is of great significance for building a resource-saving, environment-friendly agriculture, and realizing the sustainable development of agriculture [11].Rational recycling of agricultural waste and the development of ecological recycling agriculture are the key tasks of China’s agricultural development in the future [12,13]. The combining of diversified forage grain rotation planting with animal husbandry in France had good environmental and economic benefits [14]. The agricultural and animal husbandry cycle model in the Amazon basin of Brazil could reduce chemical input, improve agricultural productivity, and reduce soil degradation and deforestation [15]. The coupling of grain and rapeseed production and animal husbandry in Australia could increase the productivity of crops and livestock by 25–75% with the least external input [16]. On the whole, most studies showed that the agricultural and animal husbandry cycle model played an important role in global food security, improving farmers’ livelihoods, reducing environmental pollution, and reducing greenhouse gas emissions [17,18,19].

As an emerging indicator, nitrogen footprint (NF) is defined as the total amount of Nr (released to the environment due to resource consumption by an entity, expressed in total units of Nr [20]. Most of the previous studies have focused on the national-scale per capita NF [21,22], or the NF of food consumption [23,24]. Afterwards, NF was gradually extended and applied to agricultural systems. For example, Xue et al. [25] reported on the nitrogen footprint of double-cropping rice in southern China, Zhang et al. [26] calculated the nitrogen footprint of four agricultural products, some scholars have also studied the nitrogen footprint of vegetables [27], and some Italian scholars conducted nitrogen footprint accounting for organic pork [28]. Quantitative assessment of the NF of these agricultural systems is critical for mitigating climate change and reducing environmental pollution, while the NF studies that take into account the coupling of cropland systems and livestock systems are uncommon. In consequence, using the NF method can help policymakers understand the environmental impacts of circular farming systems and make more informed decisions for future food security and environmental protection.

In the view of life-cycle, this research focuses on crop production and pig production in the North China Plain to explore their Nr emissions and NFs. The objectives of this study were to: (1) quantify the NF for the crop-swine integrated system (IS) and the separated crop production/swine production system (SS); (2) compare the differences between IS and SS and analyze the reasons for the differences in NFs of different agricultural circulatory systems. The results can provide a scientific basis for the sustainable development model of circular agriculture.

## 2. Materials and Methods

### 2.1. Goals and Functional Units

Life cycle assessment was used to quantify the NFs of the crop-swine integrated system and separated system, and analyze the corresponding reasons. The integrated system refers to the crop-swine integrated system (IS), the separated system (SS) includes both the crop production system (SS-C) and the swine production system (SS-P). Since the effects of various forms of Nr are different, it is difficult to comprehensively evaluate the multiple effects. This study chose the eutrophication potential for nitrogen environmental impact assessment [29] to quantify the NF of two systems. The results are expressed in g N-eq per hectare per year for a certain number of pigs (g N-eq ha^−^^1^yr^−^^1^ for pigs), g N-eq per hectare per year (g N-eq ha^−^^1^yr^−^^1^), and g N-eq per year for a certain number of pigs (g N-eq yr^−^^1^ for pigs) for the IS, SS-C, and SS-P, respectively.

### 2.2. System Characteristics

A full chain analysis, including forage processing, pig rearing, and crop production of the active nitrogen for the SS and IS in the North China Plain was implied in this study.

#### 2.2.1. Separated System

**Figure 1:**
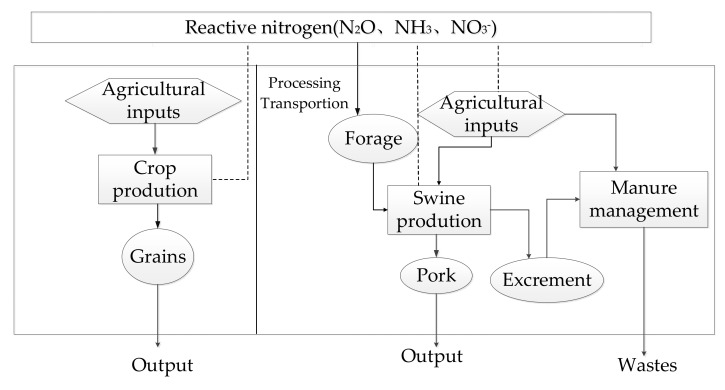
The system boundary of the separated system (SS). Notes: solid arrow denotes energy and material flow under agriculture practices, dotted arrow denotes reactive nitrogen emissions from agriculture.

The system boundary is shown in Figure 1. The system boundary of crop production included not only direct Nr emissions from farmland, but also indirect emissions from field management, which including: (1) the production, processing, transportation, and sales of agricultural inputs (such as seeds, agricultural films, fertilizers, pesticides, etc.); (2) energy consumption of mechanical operations in crop production, such as tillage, sowing, harvesting, etc.; and (3) loss of active nitrogen in farmland (such as ammonia volatilization, nitrous oxide emission, nitrate leaching). The cropping pattern in the separated system was winter wheat and summer maize. The straw and manure were returned to the field after the crop harvesting, and the chemical fertilizer was applied in two equal amounts before sowing and at the jointing stage.

Forage processing, swine rearing, and waste manure management were considered for the swine production system in the SS. For forage processing, Nr emissions were from purchased maize as the primary material for premix forage, electricity, and fossil fuel as power. The wheat bran and soybean meal used for forage processing were wastes from the local food industry and the corresponding Nr emissions were ignored. The swine rearing period was usually 180 days in this region and the Nr emitted from feeds, electricity and fossil fuel for lighting and heating; and various inputs such as vaccine, disinfectant, veterinary medicine, etc. was taken into account After 180 days of stacking during breeding, pig manure was randomly stored as solid waste and was not reused. The waste was then dumped directly onto the land, which was a common practice on many intensive pig farms. Wasted feces emissions last for one year. Due to dumping directly onto the abandoned land, the contribution of organic nitrogen contained in the remaining manure to soil nitrogen fixation was not considered. The detailed inputs and outputs for crop production and swine production were shown in Table 1 and Table 2, respectively.

#### 2.2.2. Crop-Swine Integrated System Introduction

**Figure 2:**
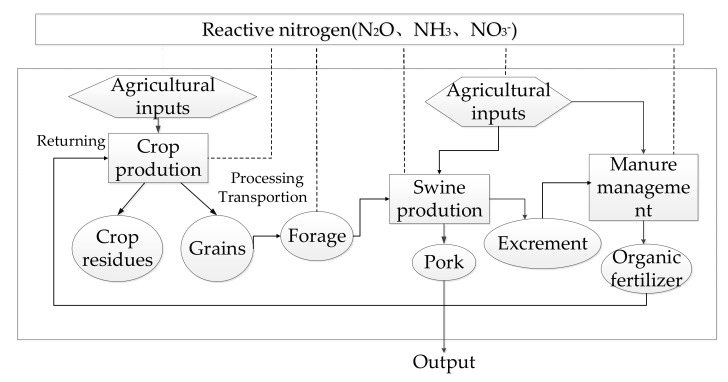
System boundary of the Crop-swine integrated system (IS). Notes: solid arrow denotes energy and material flows under agriculture practices, dotted arrow denotes reactive nitrogen emissions from agriculture.

Compared with SS, IS mainly considered the integrated system of crop planting and pig breeding (Figure 2), which including crop production, forage processing, and swine rearing subsystems. Nr emissions from each process are taken into account. The grain produced from the farmland provides primary material for premix forage for the swine production subsystem, and the livestock subsystem returns the manure to the farmland. These processes combined two separated subsystems to an integrated system. The crop residues were removed out the farmland in order to avoid interfering the results. In this study, the connection of the crop production subsystem and the pig rearing subsystem is based on the application of pig manure containing 225 kg of pure nitrogen according to 1 hectare of farmland. Since the total nitrogen content of pig manure produced by each pig is 6.06 kg, the corresponding number of pigs to 1 hectare of farmland is 37.

Compared to the SS, the IS was an optimized solution and produces different Nr emissions because of less chemical fertilizer used. The differences of Nr emissions between the two systems were from the following aspects. The Nr emissions from farmland were different due to the return of swine manure and a subsequent difference for chemical fertilizers. In the IS-P, because of different manure management procedures, the amount of Nr emissions was different for this processing. The transportation distance was different: SS-P require trucks to transport primary material for premix forage, since swine production is separated from crop production, while IS eliminate the need to transport. The average transportation distance was estimated to 200 km of this study.

### 2.3. Field Experiments

#### 2.3.1. Study Sites and Experimental Design

The experiment was initiated from 2010, and the data used in this study were from 2018 to 2020. The experimental site was located at Wuqiao Experimental Station of China Agricultural University (37°41′02″ N, 116°37′23″ E), Hebei Province, China. The long-term average annual temperature was 12.9 °C, with the annual accumulated temperature (≥0 °C) of 4862.9 °C. The average annual rainfall was 568 mm, mostly from June to August. The annual sunshine was 2724.8 h, and the frost-free period was 201 days. Classification according to the IUSS Working Group WRB 2006 [30], the soil in experimental plot was silty loam. The antecedent soil properties were 4.5 and 4 g kg^−1^ SOC, 1.41 and 1.47 g cm^−1^ bulk density, and pH 8.2, respectively for 0–10 cm and 10–20 cm depth. In this experiment, a typical winter wheat summer maize double-cropping rotation system in the North China Plain was used for research.

Summer-maize was sown in mid-June and harvested in early October, followed by winter-wheat in early October and harvested in early June of the next year. This experiment was a single factor experiment of crop straw returning and pig manure returning to the field with random block design. Each treatment had 3 blocks, a total of 6 experimental plots, and the plot area was 2 m × 2 m. According to the principle of equal nitrogen amount of 300 kg ha^−1^, 150 kg ha^−1^ nitrogen fertilizer was applied in both the winter wheat season and summer maize season. The total nitrogen contained in the harvested above-ground crop straw was equal to the harvested crop straw multiplied by the crop straw nitrogen content. The whole amount of crop straw was returned to the field, and the remaining nitrogen required was supplemented with Urea. Pig manure accounted for 75% and chemical fertilizer (Urea) accounted for 25%. The organic waste was returned to the soil before the crops were planted. When returning to the field, it was manually turned into the topsoil and mixed evenly. The other agricultural managements were the same as the local level.

To estimate Nr emissions from swine production, a large-scale farm (Jinlong Company) located in Jingxian County (37°58′ N, 115°99′ E) in Hebei Province in the North China Plain, 50 km away from Wuqiao Experimental Station of China Agricultural University, was taken as an example in this study. The farm produced 100 thousand pigs per year, and the production of live pigs adopted the form of self-breeding. On average, a pig took about 180 days from farrowing to completion. All pigs were raised in indoor piggery. The average final weight of each pig was expected to be 100 kg. Raw data were obtained from farm employers and employees by a questionnaire survey.

#### 2.3.2. Sampling

##### Determination of Nitrous Oxide Emissions

Soil N_2_O emission was collected by static rectangle chambers [31,32]. The gas was collected at 0, 12, 24, and 36 min after the static chamber was closed to calculate the emission slope. In this experiment, the gas was continuously collected every 7–10 days during the whole crop growth period. We collected gas every 1st, 3rd, 6th, and 9th days after applying nitrogen fertilizer, in order to capture the gas emission peak. If it rained suddenly, we collected the gas the day after it rained, and then collecting it every 7–10 days. The gas composition was measured by a gas chromatograph and then we calculated soil N_2_O emission flux. The total greenhouse gas emissions during the growth period of the crop are calculated by linear interpolation. The N_2_O emissions fluxes (*F*) was calculated by following equation:

(1) $$F=\frac{M}{V_{0}}\;\frac{P}{P_{0}}\frac{T_{0}}{T}H\frac{d_{c}}{d_{t}}$$

where *F* was N_2_O emissions fluxes of (mg m^−2^ min^−1^), *M* is the molar mass of N_2_O (44 g mol^−1^); *V*_0_ is the molar volume of N_2_O in the standard state; *P*_0_ and *T*_0_ mean the air pressure and temperature in the standard state; *P* and *T* are the samples in the box actual pressure and temperature; *H* is the height of the chamber; $\frac{d_{c}}{d_{t}}$ represents the slope of time regression curve of the target gas concentration in the chamber.

##### Determination of Ammonia Volatilization

The ventilation method [33] was used to collect volatized NH_3_ from winter wheat-summer maize fields. The capture of soil volatile ammonia began on the day of fertilization, and sampling was performed at 24 h intervals. In the first week, the samples were taken once a day; in the second to third weeks, the samples were taken every three days, after which the sampling interval could be extended to seven days. If it rains suddenly, we sample the day after the rain for 3 consecutive days, then return to normal frequency. The ammonia volatilization rate of the field soil was calculated by the following formula:

(2) $$S_{NH_{3}}=\frac{M}{A\times D}\times 10^{-2}$$

(3) $$NE_{NH_{3}}=\sum_{i}^{n}S_{NH3}\times D$$

where $S_{NH_{3}}$ is the ammonia volatilization rate (kg ha d^−1^); *M* is the amount of ammonia measured per sponge (NH_3_-N, mg); *A* is the cross-sectional area of the capture device (m^2^); *D* is the time of each continuous capture (d). *NE* is the total amount of ammonia volatilization (kg ha^−1^).

##### Determination of Nitrate Leaching

Soil solution extractors were used to collect soil solution at a depth of 200 cm. The sampling frequency was usually once a week. If there is irrigation or heavy rainfall, the soil solution is collected continuously for a week. After that, the sampling interval was every 3 days for two consecutive weeks. The nitrate nitrogen concentration (mg L^−^^1^) of the collected soil solution was measured using a continuous flow analyzer (CFA). The nitrate leaching amount was calculated by the following equation:

(4) $$N=\frac{\sum_{i}^{n}Ci\times Li}{S}$$

(5) $$L=\alpha \left(P+I\right)$$

where *N* is nitrate leaching amount, *Ci* is nitrate nitrogen concentration, *Li* is leakage, *S* is the area of the plot; *L* (mm) is the leakage amount, *P* (mm) is the precipitation, *I* (mm) is the irrigation amount; *α* is the replenishment coefficient, when the total rainfall for 5 consecutive days is less than or equal to 90 mm, the value of *α* is 0.1. When it is more than 90 mm and less than 250 mm, the value of α is 0.15. When it is more than or equal to 250 mm, the value of *α* is 0.2.

### 2.4. Nitrogen Footprint Calculation

#### 2.4.1. Nitrogen Footprint of Crop Production System

Indirect and direct NF were considered in this study. Indirect NF contained agricultural inputs for all processing of production, transportation, and storage. Direct NF was primary in the field, including nitrate leaching, ammonia volatilization and nitrous oxide emission:

(6) $$NF_{CPS}=NE_{N_{2}O}+NE_{NH_{3}}+NL_{NO_{3}^{-}}+\underset{i}{\sum }\left(IN_{i}\times EF_{N_{i}}\right)$$

where $NF_{CPS}$ is the total NF of the crop production system; $NE_{N_{2}O}$, $NE_{NH_{3}}$, and $NL_{NO_{3}^{-}}$ are the NF caused by N_2_O emission, ammonia volatilization, and nitrate leaching; $IN_{i}$ is the amount of its input, and $EF_{N_{i}}$ is the Nr emission factor corresponding to its input.

#### 2.4.2. Nitrogen Footprint of Swine Production System

The NF of swine production system were the sum of the NF of forage processing, swine rearing and waste management, and the formula is as follows:

(7) $$NF_{SPS}=NF_{forage\;processing}+NF_{swine\;rearing}+NF_{waste\;management}$$

where $NF_{SPS}$ is the total NF of swine production system, $NF_{forage\;processing}$ is the NF of forage processing, $NF_{swine\;rearing}$ is the NF of swine rearing, $NF_{waste\;management}$ is the NF of waste management.

#### 2.4.3. Nitrogen Footprint of Separated System

The NF of separated system were the sum of the NF of crop production subsystem and pig rearing subsystem, and the formula is as follows:

(8) $$NF_{SS}=NF_{SS-C}+NF_{SS-P}$$

where $NF_{SS}$ is the total NF of separated system, $NF_{SS-C}$ is the NF of crop production subsystem in separated system, $NF_{SS-P}$ is the NF of pig rearing subsystem in separated system.

#### 2.4.4. Nitrogen Footprint of Integrated System

The NF of integrated system were the sum of crop production subsystem in integrated system and pig rearing subsystem in integrated system, and the formula is as follows:

(9) $$NF_{IS}=NF_{IS-C}+NF_{IS-P}$$

where $NF_{IS}$ is the total nitrogen footprint of integrated system, $NF_{IS-C}$ is the NF of crop production subsystem in integrated system, $NF_{IS-P}$ is the NF of pig rearing subsystem in integrated system.

## 3. Results

### 3.1. Nitrogen Footprint of Crop Production Subsystem

Recycling waste swine manure to farmland increased the grain yield for both winter wheat and summer maize. The total grain yield of IS-C increased by 14.38% compared with that of SS-C. Direct Nr in cropland and indirect Nr caused by agricultural inputs were considered to calculate the NF of the integrated crop-swine and separated systems. The results, shown in Table 3, indicated that recycling swine manure to cropland reduced the NF per unit area by 12.02% for the crop production system, compared to that of the separation system. The performance was better for IS-C if considering the grain production, where the NF per unit grain yield reduced by 23.08% for IS-C compared to that of SS-C.

Direct NF was the main contributor for both systems, accounting 79.07% for IS-C and 78.39% for SS-C, respectively. The direct NF was 118.19 kg N-eq ha^−1^ for IS-C, which is 12.79% lower than that of SS-C. The reduction of direct NF for IS-C was mainly due to the lower direct Nr caused by NH_3_ volatilization. Due to the reduction of fertilizer consumption, the Nr caused by NH_3_ volatilization for IS-C decreased by 17.33 kg N-eq ha^−1^ compared to that of SS-C. The Nr caused by N_2_O emission was reduced by 3.33 kg N-eq ha^−1^ for IS-C as well. However, the Nr caused by nitrate leaching was increased by 6.03 kg N-eq ha^−^^1^.

**Figure 3:**
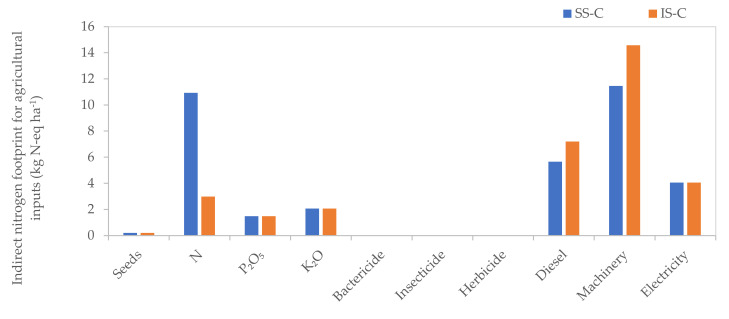
NF of agricultural inputs for SS-C and IS-C. SS-C is the crop production of separated system, IS-C is the crop production of crop-swine integrated system.

There are differences for indirect NF between the systems (Table 3). The indirect NF was 32.59 kg N-eq ha^−1^ for IS-C, which was 9.13% lower than that of SS-C. The contributions of various agricultural inputs were different for SS-C and IS-C. Mechanical was the largest contributor for both IS-C and SS-C, accounting for 31.93% and 44.75%, respectively. The second contributor was nitrogen fertilizer (30.49%) for SS-C and diesel (22.09%) for IS-C. Fertilizer input and pig manure transportation were the factors that caused the difference between the two systems. SS-C used more fertilizer than IS-C (266.17%), and IS-C consumed more diesel and machinery than SS-C (27.32%) to transport pig manure (Figure 3).

### 3.2. Nitrogen Footprint of Pig Rearing for Separated and Integrated Crop-Swine System

The NF of pig rearing subsystem including forage processing, pig growing and waste management steps. As shown in Table 4, the total NF of pig rearing system was 317.03 kg N-eq and 254.34 kg N-eq for SS-P and IS-P, respectively. Forage processing is the link from crop production to pig growing production, which is a key part in the life cycle of pig rearing system. The NF from forage processing is much higher than pig rearing and waste management, with SS-P accounting for 73.80% and IS-P accounting for 88.59%. Compound forage during forage processing contributed the largest proportion of NF in both SS-P and IS-P. Due to the combining of crop production and pig production, the pig manure produced by IS-P was returned to the field as organic fertilizer, and the maize produced by IS-C was used as the raw material of premix forage, resulting in the differences of IS-P and SS-P on maize forage input, forage transportation and waste management. Soybean meal and wheat bran were not considered for NFs in this study as they are waste during food production, however, the transportation was considered. Therefore, IS-P reduced the NF by 5.16 kg N-eq, 3.49 kg N-eq and 54.05 kg N-eq for maize forage input, forage transportation and waste management, respectively, compared to SS-P. The NF of pig growing had no difference for SS-P and IS-P, with the amount of 27.56 kg N-eq. In general, compared to separated system, pig rearing system of integrated crop-swine reduced potential environmental pollution.

### 3.3. Nitrogen Footprint of the Separated and Integrated Crop-Swine System

With the life cycle perspective, crop production, forage processing, pig growing, and waste management were contained to calculate the total NF in the crop-swine system. As shown in Table 5, the amount of total NF of SS and IS was 488.41 kg N-eq ha^−1^ and 405.11 kg N-eq ha^−1^, respectively. IS reduced NF by 17.06% compared to SS because of recycling use of maize grain and swine manure. There is no difference in pig rearing step. Correspondingly, the NF of crop production, forage processing, and waste management for IS was 12.02%, 3.69%, and 97.36% lower than that of SS. Overall, integrated crop-swine system decreased potential environment pollution and was a recommend pattern of farmer choice.

## 4. Discussions

### 4.1. Impactions of Circular Agriculture on Reactive Nitrogen of Crop Production System

The conventional agriculture is facing the challenge of food insecurity and various environmental pollutions. In order to perusing higher yield, the highly intensive practices were widely used which brought great pressure to the environment [34]. Therefore, it is urgent to find out the pathways to obtain high yield with less input. Integrated crop with swine system was an efficient attempt to mitigate the problem, which not only disposed the waste, but also replaced part of the chemical fertilizer. The input of manure decreased the consumption of N fertilizer by 199.63 kg ha^−1^, while significantly improved the outputs of the crop production. The total grain yield for IS was 14.38% higher than that of SS. The effect of manure on increasing wheat yield (27.97%) was better than that of corn (6.55%). According to the results of meta-analysis, substituting manure for fertilizer significantly increased the yield of grain crop by 5.20% and crop N uptake and nitrogen use efficiency was significantly increased by substituting manure for fertilizer by 8.80% and 10.40% for grain crop, respectively [35]. The increase in crop yields is largely due to increased uptake of nitrogen and other mineral nutrients [36]. Substituting manure for fertilizer could increase the supply of organic carbon, thereby promoting microbial fixation of bioavailable nitrogen that would otherwise be lost to the environment. At the same time, it is also necessary to consider the proportion of organic fertilizer and the total amount of fertilizer applied. The input of manure not only improved the crop yield, but also decreased Nr loss. The direct Nr loss was 135.51 kg ha^−1^ for SS-C system, and 118.19 kg ha^−1^ for IS-C. The Nr loss was decreased by 12.79% for IS-C compared to SS-C, in which N_2_O was decreased by 46.94%, NH_3_ was decreased by 16.15%, while the NO_3_^−^ was increased by 137.51%. The primarily reason for the decrease of active N of IS-C was the reduction of chemical fertilizer.

Whether livestock manure increases N_2_O emissions compared with chemical N fertilizers is controversial [37,38,39,40]. In the study by the peri-urban zone of Zhejiang [41], the application of organic fertilizers partially replacing chemical fertilizers significantly reduced N_2_O emissions, by an average of 34.10%, which is consistent with the results of this study. However, there are also studies showing that the application of fertilizers can significantly increase N_2_O emissions, with an average increase of 32.70%, compared with the application of chemical nitrogen fertilizers alone [37]. On one hand, compared to chemical nitrogen fertilizers alone, manure application provides energy for microbial activity, that is, unstable organic carbon compounds, which stimulate N_2_O production of nitrifiers and denitrifiers [42]. On the other hand, Fertilization increases the availability of C and N substrates, which in turn enhances microbial activity and O_2_ consumption, and promotes denitrification, thereby increasing N_2_O emissions [38]. However, it should be noted that the effect of manure on soil N_2_O emissions depends on soil texture, manure type, climate, fertilization method and pH, of which pH is a particularly important factor. Application of manure to replace chemical fertilizers significantly increased N_2_O emissions in acidic soils (pH < 6.5), but not in neutral and alkaline soils [37]. The North China Plain, where this study is located, is the region where the soil pH is alkaline, which just confirms the result that pig manure can reduce soil N_2_O in this study. Ammonia volatilization is the main contributor of reactive nitrogen in farmland, which is consistent with the conclusion of Li et al. [43]. In this study, the application of pig manure can reduce soil ammonia volatilization by 16.2%, which is consistent with previous studies [44,45]. Fenn and Hossner [42] reported that NH_3_ volatilization is driven by an equilibrium between NH_4_^+^ and NH_3_ in soil solutions, which was influenced by pH. Previous studies have shown that the application of pig manure significantly reduces soil pH for the duration of fertilization [45]. In addition, studies have also shown that manure application can delay soil NH_3_-N emission, because the rate of conversion of N into NH_4_^+^ in manure is slower than that of inorganic N, which limits the volatilization of NH_3_ [46]. The results of the present study showed that pig manure increased NO_3_^−^leaching, which was inconsistent with Guo et al. [47] finding that replacing fertilizer with pig manure and chicken manure reduced nitrogen leaching. The reason is that the substitution of organic nitrogen fertilizers for chemical nitrogen fertilizers can reduce nitrogen leaching because of slow mineralization of organic nitrogen and relatively low nitrogen availability [48]. The possible reason for the increase in nitrate leaching in this experiment is that after years of continuous fertilization, the soil nitrogen content of IS-C is higher than that of SS-C, so IS-C is more likely to cause nitrate leaching than SS-C.

### 4.2. Impactions of Nitrogen Footprint on Separated and Crop-Swine Integrated System

The study by Chen et al. [49] showed that the total Nr emission from China’s agro-ecosystem in 2011 was 14.40 Tg N, chemical nitrogen fertilizers ranked first with 41.40% of total emissions, followed by manure management with 27.60%. Once lost to the environment, these Nr species can trigger various environmental changes, such as smog, acid rain, stratospheric ozone depletion, and increased greenhouse effect, with negative impacts on ecosystems and humans [50]. Previous results showed that 1 kg live-weight pig production generated 59 g Nr emissions with large regional variations [51]. Based on the results of extensive organic methods of the Italian Mora Romagnola breed [28] had shown that the total amount of Nr released was about 40 kg per pig (live weight). The SS-P and IS-P NFs of this study were 317.03 kg N-eq ha^−1^and 254.34 kg N-eq ha^−1^ (37 pigs), respectively, which were converted to 8.57 and 6.87 per head, slightly higher than the results of previous studies, which may be due to inconsistent system boundaries and timeframe studies.

Crop-livestock systems (local integration of crops and livestock systems) are often considered as a way to improve agricultural system attributes such as productivity, resource use efficiency. The coupling between the crop production system and the pig production system allows them to obtain the resources they need, such as manure and feed, which may replace some of the purchased fertilizer and premix inputs. In addition, the coupling of the crop-livestock system is a means of tackling nitrogen pollution in China [52,53]. Results showed that farms in cooperative crop-livestock systems had lower Nr emissions (21–40%) compared to those in decoupled specialized livestock systems [54]. In this study the amount of total NF of SS and IS was 485.37 kg N-eq ha^−1^ and 402.07 kg N-eq ha^−1^, respectively. By coupling the crop production and swine production systems, the maize produced by the system was directly used as feed and the pig manure was used for returning to the field, which could reduce the nitrogen emission of the system by 80 kg N-eq ha^−1^. Among them, the two important links to reduce the NF were that the return of pig manure to the field reduced the input of nitrogen fertilizer on the farmland and the maize as part of the feed reduced the amount of maize purchased outside the system.

### 4.3. Limitations and Further Research

In this study, some links were not considered, such as the construction of pig houses, because the construction of pig houses was not the difference between the two systems, and the proportion was small, so it was ignored. In addition, most of the parameters used were from the Ecoinvent, and its data were the global average data, which might cause some inaccuracies in the calculation of the North China Plain. Furthermore, the leakage volume estimation of nitrate leaching was not accurate. As a result of altered surface soil properties, the leakage volume of pig manure returning to the field and straw returning to the field should be different. According to our results, the integrated system had a great potential for manure management although it reduced Nr emissions compared to the separated system. Compared to direct application as a fertilizer, anaerobic digestion was a more efficient method of manure management and treatment because volatile ammonia was stabilized as non-volatile nitrate in the digestate [55]. The solid residue (digestate) produced during the digestion process contains carbon, organic nitrogen and nitrates, could be promoted as an organic fertilizer. During anaerobic digestion, biogas was also produced, which could be burned for heat and power generation [56]. Therefore, our further research may consider lengthening the circulation chain and adding more circulation systems such as biogas production systems, to compare their effects on reducing Nr emissions.

## 5. Conclusions

In China, we are facing the dilemma that nitrogen fertilizers must be used to ensure food security, and agricultural wastes with large quantities and negative environmental impacts must be utilized. Therefore, coupling the crop system and the breeding system is an excellent choice to solve these two problems. We used the LCA perspective to compare the differences in nitrogen footprints of the separated and integrated systems. Our results demonstrate that the coupled system can reduce the nitrogen footprint while increasing the output, in which the yield is increased by 12.57%, the nitrogen footprint of the coupled system is 17.06% less than that of the separation system, and the two subsystems are also reduced by 12.02% and 19.78%, respectively. The difference between the two systems comes from three differences, the replacement of fertilizers with pig manure, the transportation of forage and the management of pig manure. Among them, the recycling of manure and the reduction of field ammonia volatilization due to the substitution of chemical fertilizers are the most dominant reasons.

As a link between crop planting and pig breeding, the IS not only saves the input of chemical fertilizers, but also promotes the utilization of manure, increases crop yield and decreases environmental pollution, which is a green and sustainable development model that is worth promoting. This research is a basic research on the application of different circular agriculture models, and clarifies the list of material energy consumption and key parameters of the circular agricultural models, and provides a scientific basis for the sustainable development model of circular agriculture.

## Figures and Tables

**Table 1:** Inputs and outputs for crop production, SS-C is the crop production system of separated system and IS-C is the crop production subsystem of integrated system.

Item	SS-C	IS-C	Units	Emission Factor	Units
Inputs					
Wheat seed	262.50	262.50	kg ha^−1^	0.76	g N-eq kg^−1^
Maize seed	15.00	15.00	kg ha^−1^	0.76	g N-eq kg^−1^
N	274.63	75.00	kg ha^−1^	39.82	g N-eq kg^−1^
P_2_O_5_	52.00	52.00	kg ha^−1^	28.35	g N-eq kg^−1^
K_2_O	248.00	248.00	kg ha^−1^	8.32	g N-eq kg^−1^
Bactericide	2.63	2.63	kg ha^−1^	3.53	g N-eq kg^−1^
Insecticide	1.35	1.35	kg ha^−1^	3.53	g N-eq kg^−1^
Herbicide	1.50	1.50	kg ha^−1^	4.49	g N-eq kg^−1^
Diesel for tillage	20.40	20.40	kg ha^−1^	102.98	g N-eq kg^−1^
Diesel for sowing	15.00	15.00	kg ha^−1^	102.98	g N-eq kg^−1^
Diesel for harvesting	9.75	9.75	kg ha^−1^	102.98	g N-eq kg^−1^
Diesel for manure management	0.00	15.00	kg ha^−1^	102.98	g N-eq kg^−1^
Machinery for tillage	40.80	40.80	kg ha^−1^	104.30	g N-eq kg^−1^
Machinery for sowing	30.00	30.00	kg ha^−1^	104.30	g N-eq kg^−1^
Machinery for harvesting	19.50	19.50	kg ha^−1^	104.30	g N-eq kg^−1^
Machinery for manure management	0.00	30.00	kg ha^−1^	104.30	g N-eq kg^−1^
Electricity	1087.50	1087.50	Kwh ha^−1^	3.72	g N-eq Kwh^−1^
Outputs					
Wheat grain	6381.09 ± 794.59	8165.96 ± 1193.56	kg ha^−1^		
Maize grain	11,082.77 ± 1066.06	11,808.46 ± 1015.62	kg ha^−1^		

**Table 2:** Inputs and outputs for swine production, SS-P is the pig production system of separated system and IS-P is the pig production subsystem of integrated system.

Items	SS-P	IS-P	Units	Emission Factor	Units
**Forage processing**					
Maize	183.38	0.00	kg head^−1^	0.76	g N-eq kg^−1^
Soybean meal	70.04	70.04	kg head^−1^	0.76	g N-eq kg^−1^
Wheat bran	49.07	49.07	kg head^−1^	0.76	g N-eq kg^−1^
Compound forage	315.98	315.98	kg head^−1^	17.76	g N-eq kg^−1^
Microelement	14.16	14.16	kg head^−1^	17.34	g N-eq kg^−1^
Transportation	200.00	200.00	km	2.57	g N-eq t^−1^ km^−1^
Electricity	0.02	0.02	kWh head^−1^	3.72	g N-eq kWh^−1^
Fossil fuel	0.02	0.02	kg head^−1^	102.98	g N-eq kg^−1^
**Swine rearing**					
Water	2634.26	2634.26	kg head^−1^	2.60 × 10^−4^	g N-eq kg^−1^
Concentrate feed	19.49	19.49	kg head^−1^	21.57	g N-eq kg^−1^
Premixed feeds	13.49	13.49	kg head^−1^	17.76	g N-eq kg^−1^
Electricity	20.11	20.11	kWh head^−1^	3.72	g N-eq kWh^−1^
Coal	8.65	8.65	kg head^−1^	2.70 × 10^−3^	g N-eq kg^−1^
Vaccine	37.11	37.11	g head^−1^	80.35	g N-eq kg^−1^
Disinfectant	1542.03	1542.03	g head^−1^	0.17	g N-eq kg^−1^
Veterinary medicine	75.10	75.10	g head^−1^	80.35	g N-eq kg^−1^
**Outputs**					
Pork	100 kg head^−1^				
Urine	314 kg head^−1^				
Feces	66.80 kg dry head^−1^				

**Table 3:** NF of crop production subsystem. SS-C is the crop production of separated system, IS-C is the crop production of crop-swine integrated system.

Item	Units	SS-C	IS-C	Statistical Analysis
Wheat yield	kg ha^−1^	6381.09 ± 794.59	8165.96 ± 1193.56	*
Maize yield	kg ha^−1^	11,082.77 ± 1066.06	11,808.46 ± 1015.62	-
Total yield	kg ha^−1^	17,463.86 ± 1590.94	19,974.42 ± 1789.15	*
Indirect NF	kg N ha^−1^	35.86	32.59	
Direct NF	kg N ha^−1^	135.51 ± 32.11	118.19 ± 31.77	-
including				
N_2_O	kg N ha^−1^	7.10 ± 3.17	3.77 ± 2.85	-
NH_3_	kg N ha^−1^	124.02 ± 29.98	104.00 ± 23.34	-
NO_3_^−^	kg N ha^−1^	4.39 ± 0.14	10.42 ± 7.70	-
NF per unit area	kg N-eq ha^−1^	171.37	150.77	
NF per unit grain yield	kg N-eq t^−1^	98.13	75.48	

Note: * represents a significant difference at the *p* = 0.05 level. -represents no significant difference at *p* = 0.05 level.

**Table 4:** NF of pig rearing system. SS-P is the pig rearing of separated system, IS-P is the pig rearing of integrated crop-swine system.

Items	Units 37 Head Pigs^−1^	SS-P	IS-P
**Forage processing**			
Maize	g N-eq	5156.65	0.00
Soybean meal	g N-eq	0.00	0.00
Wheat bran	g N-eq	0.00	0.00
Compound forage	g N-eq	207,605.01	207,605.01
Microelement	g N-eq	9086.95	9086.95
Transportation	g N-eq	12,029.07	8542.21
Electricity	g N-eq	2.76	2.76
Fossil fuel	g N-eq	76.21	76.21
**Pig rearing**			
Water	g N-eq	25.36	25.36
Concentrate feed	g N-eq	15,552.57	15,552.57
Premixed feeds	g N-eq	8863.19	8863.19
Electricity	g N-eq	2771.49	2771.49
Coal	g N-eq	0.86	0.86
Vaccine	g N-eq	110.33	110.33
Disinfectant	g N-eq	9.98	9.98
Veterinary medicine	g N-eq	223.28	223.28
**Waste management**	g N-eq	55,521.14	1468.11
**Total**	kg N-eq	317.03	254.34

**Table 5:** NF of the separated and integrated crop-swine system.

Items	Units	SS	IS
Crop Production	kg N-eq ha^−1^	171.37	150.77
Forage processing	kg N-eq ha^−1^	233.96	225.31
Pig rearing	kg N-eq ha^−1^	27.56	27.56
Waste management	kg N-eq ha^−1^	55.52	1.47
Total	kg N-eq ha^−1^	488.41	405.11

## Data Availability

Not applicable.
